# High-Throughput Absolute Quantification Sequencing Revealed Osteoporosis-Related Gut Microbiota Alterations in Han Chinese Elderly

**DOI:** 10.3389/fcimb.2021.630372

**Published:** 2021-04-30

**Authors:** Muhong Wei, Can Li, Yu Dai, Haolong Zhou, Yuan Cui, Yun Zeng, Qin Huang, Qi Wang

**Affiliations:** ^1^ MOE Key Lab of Environment and Health, Department of Epidemiology and Biostatistics, School of Public Health, Tongji Medical College, Huazhong University of Science and Technology, Wuhan, China; ^2^ Department of Nuclear Medicine, Union Hospital, Tongji Medical College, Huazhong University of Science and Technology, Wuhan, China; ^3^ Department of Medical Record Statistics, Wuhan NO.1 Hospital, Wuhan, China; ^4^ Department of Rehabilitation Medicine, Union Hospital, Tongji Medical College, Huazhong University of Science and Technology, Wuhan, China

**Keywords:** osteoporosis, bone mineral density, microbiome, 16S ribosomal RNA sequencing, absolute quantification

## Abstract

**Objective:**

Accumulative evidence suggests that gut microbiota play an important role in bone remodeling and hence bone health maintenance. This study aimed to explore the association of gut microbiota with the risk of osteoporosis and to identify potential disease-related taxa, which may be promising targets in osteoporosis prevention and treatment in the future.

**Methods:**

Absolute quantification 16S ribosomal RNA gene sequencing was used to detect absolute and relative abundances of gut microbiota in 44 patients with osteoporosis and 64 controls. In combination with one of our previous studies, a total of 175 samples were involved in the relative abundance analysis.

**Results:**

Compared with the controls, the patients with osteoporosis had higher absolute and relative abundances of Bacteroidetes phylum, and *Bacteroides* and *Eisenbergiella* genera. The absolute abundances of *Clostridium_XlVa*, *Coprococcus*, *Lactobacillus*, and *Eggerthella* genera increased, and that of the *Veillonella* genus decreased in the osteoporosis group. As for relative abundance, that of the *Parabacteroides* and *Flavonifractor* genera increased, whereas that of the *Raoultella* genus decreased in the osteoporosis group. Controlling for potential confounders, the associations of *Clostridium_XlVa*, *Coprococcus*, and *Veillonella* genera with the risk of osteoporosis did not maintain significance. Ridge regression analysis suggested that *Bacteroides* is associated with reduced bone mineral density (BMD) and T-score at lumbar spines, and *Anaerovorax* is associated with increased BMD at the femoral neck. Functional predictions revealed that 10 Kyoto Encyclopedia of Genes and Genomes pathways were enriched in the osteoporosis group.

**Conclusions:**

Gut microbiota compositions may contribute to the risk of osteoporosis. Several specific taxa and functional pathways are identified to associate with reduced bone density, thus providing epidemiologic evidence for the potential role of aberrant gut microbiota in osteoporosis pathogenesis.

## Introduction

The human body is populated by trillions of microorganisms, and a vast majority of which consists of more than 1000 species of microbes that inhabit the gastrointestinal tract (Falony et al., 2016). Gut microbiota is crucial in maintaining human health, promoting the defensive responses to pathogen invasion and regulating immunity in the host (Carding et al., 2015). Alternated gut microbiota compositions have been linked with a range of chronic clinical conditions including obesity, diabetes, heart disease, and Alzheimer’s disease (Bordalo Tonucci et al., 2017; Jiang et al., 2017; Tang et al., 2017; Torres-Fuentes et al., 2017).

Emerging evidence also suggests an association between gut microbiota and bone health. Despite inconsistencies, previous studies have reported that germ-free mice show changed bone mass compared with conventionally raised ones (Sjögren et al., 2012; Schwarzer et al., 2016). Moreover, oral antibiotics capable of regulating gut microbiota compositions affect bone mass (Cox et al., 2014; Guss et al., 2017). In addition, animal and human studies have demonstrated the benefit of probiotics, i.e., reducing bone loss (Britton et al., 2014; Nilsson et al., 2018). Gut microbiota may affect bone remodeling by regulating nutrient (e.g., calcium) absorption in the intestinal tract, thereby regulating host immune system and functions indirectly on bones mediated by systematic circulation-translocating microbes and molecular products (e.g., serotonin and short chain fatty acids) of microbiota (Yan et al., 2016; D’Amelio and Sassi, 2018).

Osteoporosis is a common bone disorder characterized by reduced bone mineral density (BMD), altered bone microstructure, and increased fracture risk (Locantore et al., 2020). One important complication of osteoporosis is fragility fracture, which easily occurs after minor injuries and possibly results in enormous distressful events (body pains, physical function impairments, mental depressions, and even mortality) in patients (Giangregorio et al., 2014). At present, osteoporosis therapeutics mainly depends on medications of reducing bone resorption, and/or enhancing bone formation, with potential safety and tolerance problems during long-term treatment (Khosla and Hofbauer, 2017; Locantore et al., 2020). Studies on gut microbiota composition identify disease-related microbial biomarkers and may provide new directions for osteoporosis screening, diagnosis, and treatment in the future.

The presence of high-throughput sequencing technology has dramatically accelerated association studies of gut microbiota and human well-being, enabling researchers to profile microbial community compositions and functions in a high-resolution and culture-independent pattern (Franzosa et al., 2015). So far, several studies have been conducted using 16S ribosomal RNA (rRNA) gene sequencing and linked microbial alterations to varied bone mass in human beings. In one of our previous studies, the relative abundance of gut microbiota differed at several levels (phylum, genus, etc.) among Chinese elderly individuals with different bone densities (Li et al., 2019). Similarly, Das et. Al. and Wang et al. observed osteoporosis-related taxa-specific changes in gut microbiota profiles (Wang et al., 2017; Das et al., 2019). In those studies, microbial relative abundance was detected and compared between groups of different BMDs. However, the relative measurements are inadequate to reveal exact disease-related microbial alterations in case of substantial variations in microbial loads among samples (Vandeputte et al., 2017). Thus, associations of absolute compositions of gut microbiota and osteoporosis risk must be further investigated.

In this study, we adopted absolute quantification 16S rRNA gene sequencing to determine the absolute and relative abundance measurements of gut microbiota simultaneously, identified the key disease-related microbiota taking both relative and absolute profiling into account, and hence explored potential roles of gut microbiota in osteoporosis pathogenesis.

## Methods

### Participant Enrollment and Data Collection

This study was approved by the Institutional Review Board of Tongji Medical College, Huazhong University of Science and Technology. Written informed consents were obtained before the study. All participants were recruited at Union Hospital of Tongji Medical College in Wuhan City from 2018 to 2019. Adults older than 60 years or postmenopausal women with natural menopause were included in this study. Individuals taking antibiotics or hormones within the past month before stool collection were excluded. Participants with disease history of hyperthyroidism or hypothyroidism, and prevalent gastrointestinal, renal, or osteoarthritis diseases were also excluded. Women with hysterectomy and ovariectomies were excluded. Dual-energy X-ray absorptiometry (Lunar Prodigy, GE, USA) was applied to measure BMD at skeleton sites of the lumbar spine (L1–L4) and femoral neck of each participant. A T-score of ≤ −2.5 at any skeleton site was designated as prevalent osteoporosis. Finally, 44 patients with osteoporosis and 64 controls were involved in our analysis. Demographics data (sex, age, body weight, and height); cigarette smoking, alcohol drinking, and dietary habits; and disease and medication histories were collected before BMD examinations by trained investigators. Body mass index (BMI) was calculated as weight (kg) divided by the square of height (m).

### Stool Sample Collection and Microbiota Sequencing

Fresh stools were collected in sterile tubes transported with ice packs and stored at −80°C until laboratory detection within 3 months. The absolute quantification of 16S rRNA gene sequencing was conducted by Shanghai Genesky Biotechnologies Inc., Shanghai, China. Genomic DNA was extracted with FastDNA™ SPIN Kit (mpbio, California, USA) according to the manufacturer’s instructions. The integrity of genomic DNA was detected through agarose gel electrophoresis. NanoDrop2000 (Thermo Fisher Scientific, Massachusetts, USA) and Qubit3.0 spectrophotometers (Thermo Fisher Scientific, Massachusetts, USA) were used to examine the concentration and purity of DNA extracts. The spike-in sequences with identical conserved regions to natural 16S rRNA genes and variable regions replaced by random sequences with approximately 40% GC content were artificially synthesized. The spike-in sequences with known gradient copy numbers were added to the sampled DNA pools, functioning as internal standard, and allowed the absolution quantification across samples. The V3-V4 hypervariable regions of microbial 16S rRNA gene and spike-in sequences were amplified with a forward primer (Illumina adapter sequence 1 + CCTACGGGNGGCWGCAG) and reverse primer (Illumina adapter sequence 2 + GACTACHVGGGTATCTAATCC). PCR amplification was achieved on an ABI 2720 thermal cycler (Thermo Fisher Scientific, Massachusetts, USA) with a TopTaq DNA polymerase kit (Transgen, BeiJing, China). After library quantification, pooling and quality check, all samples were sequenced on the Illumina NovaSeq 6000 platform (Illumina, California, USA) with the NovaSeq 6000 SP Reagent Kit (500 cycles) (Illumina, California, USA) using the 2×250 bp paired-end method.

Raw data from the Illumina platform were then processed as described in our previous study (Li et al., 2019). Only sequences of >100 bp and those with an average score of >20 were included for further analysis. Operational taxonomic units (OTUs) were generated by clustering the clean sequences at a similarity level of 97%, and chimeras were removed by USEARCH (v10). The spike-in sequences were filtered out for read counting. The standard curve of spike-in sequences was generated for each sample, and the sequenced microbial DNA was quantified and estimated in reference to the representative standard curve (Jiang et al., 2019). Taxonomic annotation was performed at a confidence threshold of 80% by Mothur (v1.41.1) with the command classify.seqs based on the RDP (v11.5) database.

### Statistical Analysis

All statistical analyses were performed using R version 3.4.3, SPSS version 22 and GraphPad Prism version 5.01. Microbial alpha diversity was assessed with Chao1 for community richness, and with Shannon and Simpson for community diversity. Principal coordinates analysis (PCoA) was performed using weighted-UniFrac distance matrix to assess microbial beta diversity at the OTU level. Permutational multivariate analysis of variance (PERMANOVA) was performed using the Adonis () function in the R package vegan with 9999 permutations to evaluate the between-group differences of microbial communities.

The Wilcoxon rank sum test was used for between-group comparisons of absolute and relative taxon abundances at the phylum and genus levels; only shared taxa in over 20% of samples with a relative abundance of >0.01% were included. As to the microbial relative abundance analysis, data from our previous study using a same laboratory sequencing platform were combined. Multi-covariate adjusted generalized linear model was then performed to analyze the dependency of the risk of osteoporosis on the specific taxa of significantly different abundance between the case and control groups with a presumed negative binomial distribution: Taxon ~ osteoporosis-status + confounders.

Spearman correlation analysis was performed to explore the correlations between microbial absolute abundance and BMD measurements, including BMDs, and T-scores. A penalized regression approach (ridge regression analysis) was performed to detect the effect of microbial taxa on BMD measurements adjusted for age, BMI, sex, smoking, alcohol drinking, coffee drinking, and dietary habits.

Biological functions of osteoporosis-related gut microbiota were explored on the basis of Kyoto Encyclopedia of Genes and Genomes (KEGG) using Phylogenetic Investigation of Communities by Reconstruction of Unobserved States (PICRUSt). Between-group differences in functional pathways indicated by taxa variations were assessed using Welch’s t-test. A *P* value of <0.05 was considered statistically significant.

## Results

### General Characteristics of the Participants

As shown in **Supplementary Table 1**, data of 108 individuals (44 osteoporosis cases and 64 controls) were involved in the absolute abundance analysis, and data of 175 individuals (74 osteoporosis cases and 101 controls) were included in the relative abundance analysis. The patients with osteoporosis had lower BMI than the controls. The proportions of women and fracture history were comparatively higher in the osteoporosis group than the control. Between-group differences of age, smoking, alcohol drinking, coffee drinking and dietary habits did not reach the significance level.

### Between-Group Microbial Diversity Comparisons

**Figure 1:**
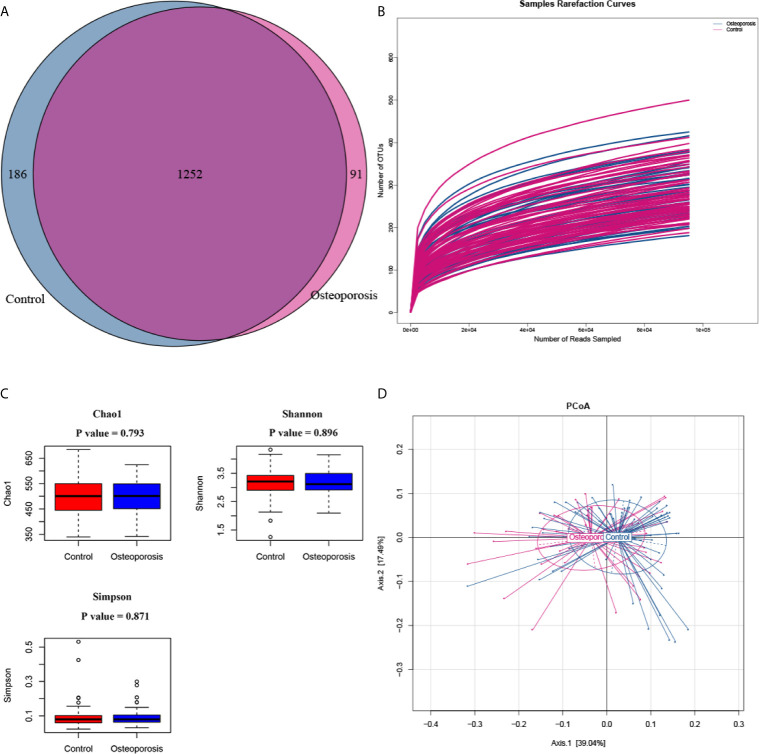
Between-group microbial diversity comparisons. **(A)** Venn diagram showing the unique and shared operational taxonomic units in the osteoporosis (case) and control groups. **(B)** Rarefaction curves for detected samples. The abscissa and ordinate represent the number of sampling sequences and the corresponding number of operational taxonomic units (OTUs), respectively. **(C)** Between-group comparisons of the alpha diversity of gut microbiota. Each box plot represents the median, interquartile range, minimum, and maximum values. *P* values were calculated by using the Wilcoxon rank sum test. **(D)** Principle coordinate analysis (PCoA) results of bacterial beta diversity based on the weighted UniFrac distance matrix. Each dot represents one sample. The control and osteoporosis groups are illustrated in blue and red colors, respectively. The two principal coordinates explain 39.04% and 17.49% of the total variance, respectively.

After low-quality, short, ambiguous and singleton reads were excluded, a total of 26,087,887 clean reads (constituting 1538 OTUs) sequenced from 108 samples were left for further analysis. The proportion of spike-in reads to total reads of each sample ranged from 12.32% to 37.00%. After spike-in reads clustered OTUs were removed, 1529 remained and the number of generated OTUs ranged from 221 to 537 per sample. The Venn diagram showed that 1252 OTUs were shared across the two groups (**Figure 1A**). The rarefaction curves for all samples were all near to saturation, indicating that the sequencing work was adequate with a few missed genes (**Figure 1B**). **Figure 1C** shows the alpha-diversity indicators for the gut microbiota in osteoporosis cases and controls. Significant between-group differences were not observed among the alpha-diversity indices (Chao1, Shannon, and Simpson). PCoA results based on weighted Unifrac matrix clustering data of microbial beta diversity among the 108 samples are shown in **Figure 1D**. PERMANOVA test results revealed achieving-significance between-group differences of microbial beta diversity (R^2^ = 0.033, *P* value = 0.022).

### Associations Between Phylum- and Genus-Level Microbial Compositions and Risk of Osteoporosis

**Figure 2:**
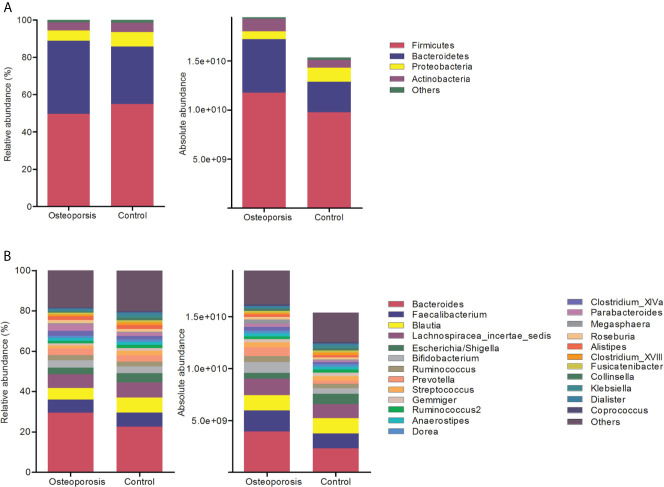
Relative and absolute abundances of the major taxa at phylum **(A)** and genus **(B)** levels in the osteoporosis and control groups.

**Figure 3:**
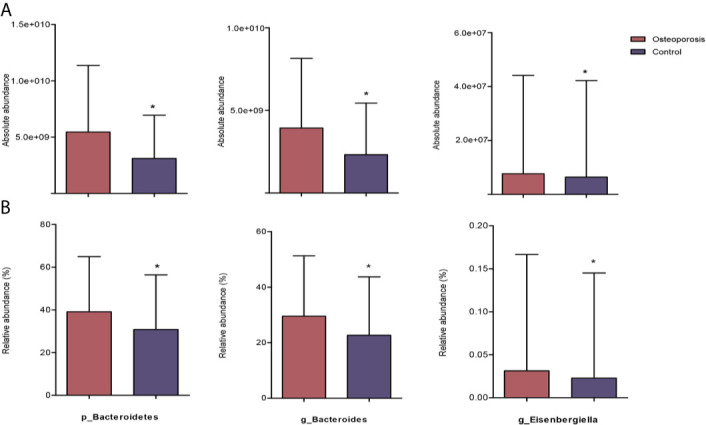
Reaching-significance of different taxa between osteoporosis and control groups with respect to absolute and relative abundances. p, phylum; g, genus; **(A)** indicates the differential analysis of absolute profiling; **(B)** indicates the differential analysis of relative profiling. ^*^0.01 ≤ *P* value < 0.05.

**Figure 4:**
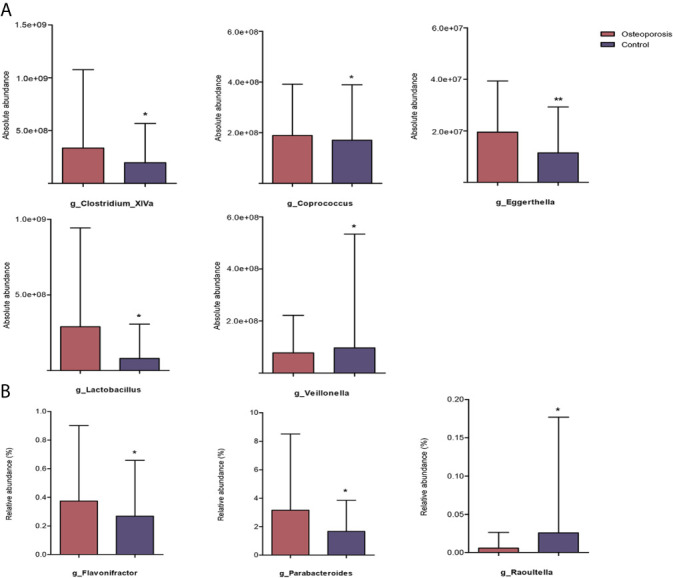
Reaching-significance of different taxa between osteoporosis and control groups with respect to either absolute or relative abundance. p, phylum; g, genus; **(A)** indicates the differential analysis of absolute profiling; **(B)** indicates the differential analysis of relative profiling. ^*^0.01 ≤ *P* value < 0.05; ^**^ *P* value < 0.01.

Results of relative abundance and absolute copy numbers of the phyla and genera in the gut microbiota among all participants are shown in **Figure 2**. Phyla Fimicutes, Bacteroidetes, Proteobacteria, and Actinobacteria dominated among all participants (**Figure 2A**). Compared with the relative and absolute abundances of the Bacteroidetes phylum in the controls, that in the osteoporosis patients was higher (**Figures 3A, B**). A total of 10 genera differed in relative and/or absolute abundances between the two groups. Specifically, the *Clostridium_XlVa*, *Coprococcus*, *Lactobacillus* and *Eggerthella* genera were enriched and *Veillonella* decreased in patients with osteoporosis referring to absolute abundance quantifications (**Figure 4A**). Compared with the controls, the patients with osteoporosis had reduced *Raoultella* and elevated *Parabacteroides* and *Flavonifractor* referring to relative abundance quantifications (**Figure 4B**). The relative and absolute abundances of *Bacteroides* and *Eisenbergiella* were higher in the osteoporosis group than the control (**Figures 3A, B**).

As shown in **Table 1**, after controlling for potential confounders, all the above-mentioned associations remained significant, except that of the absolute abundances of *Clostridium_XlVa*, *Coprococcus*, and *Veillonella* and risk of osteoporosis.

**Table 1:** Results of association analysis for absolute abundance of gut microbiota at phylum and genus levels and risk of osteoporosis.

Taxa	Mean abundance		Ratio^a^	*P* value^b^
	Osteoporosis(n = 44)	Control(n = 64)		
p_Bacteroidetes	5.45E+09	3.10E+09	1.76	0.003
g_Bacteroides	3.93E+09	2.31E+09	1.70	0.002
g_Clostridium_XlVa	3.34E+08	1.95E+08	1.71	0.070
g_Coprococcus	1.89E+08	1.71E+08	1.11	0.476
g_Lactobacillus	2.90E+08	8.00E+07	3.62	<0.001
g_Veillonella	7.77E+07	9.64E+07	0.81	0.117
g_Eisenbergiella	7.66E+06	6.41E+06	1.19	<0.001
g_Eggerthella	1.95E+07	1.14E+07	1.71	0.041

p, phylum; g, genus.

a Ratio of the mean absolute abundance of gut microbiota in the osteoporosis (case) group to that in the control group.

b P value was calculated using generalized linear model (GLM) adjusted for age, body mass index, sex, smoking, alcohol drinking, coffee drinking and dietary habits.

### Correlations Between Gut Microbiota Compositions and BMD Measurements

The results of Spearman’s correlation analysis for correlations between the absolute quantification indices for gut microbiota composition and BMD measurements are presented in **Table 2**. The Fusobacteria phylum showed negative correlation with T-score at femoral neck. The *Anaerovorax* and *Lachnospira* genera are related to BMD positively, whereas *Coprobacillus*, *Erysipelotrichaceae_incertae_sedis*, *Intestinibacter*, *Lachnospiracea_incertae_sedis*, and *Terrisporobacter* are negatively correlated with BMD at femoral neck. *Weissella* are positively linked with BMD and T-score, whereas *Bacteroides*, *Cetobacterium*, *Eggerthell*a, *Fusobacterium*, and *Megasphaera* are negatively related to bone density at lumbar spines. We also demonstrated negative associations of *Clostridium_XlVa*, and *Veillonella* with the BMD and T-score at lumbar spines and femoral neck.

**Table 2:** Spearman’s correlation estimates for gut microbial taxa and BMD measurements at phylum and genus levels.

	LS1–4 BMD	LS1–4 T-score	FN BMD	FN T-score
p_Fusobacteria	-0.181	-0.191^*^	-0.054	-0.078
g_Anaerovorax	0.158	0.107	0.255^**^	0.188
g_Bacteroides	-0.182	-0.194^*^	-0.180	-0.176
g_Cetobacterium	-0.172	-0.191^*^	-0.019	-0.020
g_Clostridium_XlVa	-0.219^*^	-0.208^*^	-0.294^**^	-0.246^*^
g_Coprobacillus	-0.112	-0.093	-0.202^*^	-0.146
g_Eggerthella	-0.207^*^	-0.209^*^	-0.152	-0.130
g_Erysipelotrichaceae_incertae_sedis	-0.094	-0.070	-0.206^*^	-0.164
g_Fusobacterium	-0.189^*^	-0.190^*^	-0.079	-0.090
g_Intestinibacter	-0.063	-0.056	-.199^*^	-0.190^*^
g_Lachnospira	0.064	0.073	0.178	0.197^*^
g_Lachnospiracea_incertae_sedis	-0.097	-0.108	-0.197^*^	-0.201^*^
g_Megasphaera	-0.208^*^	-0.215^*^	-0.163	-0.146
g_Terrisporobacter	-0.113	-0.093	-0.204^*^	-0.177
g_Veillonella	-0.249^**^	-0.241^*^	-0.217^*^	-0.217^*^
g_Weissella	0.252^**^	0.259^**^	-0.009	0.010

LS1–4, lumbar spines 1–4; BMD, bone mineral density; FN, femoral neck; p, phylum; g, genus.

Estimates were expressed as correlation coefficient, and statistical significance is indicated by ^*^0.01 ≤ P value < 0.05, and ^**^P value < 0.01.

With age, BMI, sex, smoking, alcohol drinking, coffee drinking, and dietary habits controlled, ridge regression analysis showed that the BMD and T-score at lumbar spines decreased in response to the increase in the absolute abundance of the *Bacteroides* genus, whereas the BMD at the femoral neck increased with the increase in the *Anaerovorax* genus (**Table 3**).

**Table 3:** Estimates for associations between gut microbial taxa and BMD measurements revealed by ridge regression analysis.

	LS1–4 BMD	LS1–4 T-score	FN BMD	FN T-score
p_Fusobacteria	-0.047	-0.258	0.063	0.346
g_Anaerovorax	0.036	0.220	0.063^**^	0.008
g_Bacteroides	-0.057^*^	-0.440^*^	-0.038	-0.007
g_Cetobacterium	0.010	0.010	0.045	0.004
g_Clostridium_XlVa	-0.033	-0.225	-0.035	-0.005
g_Coprobacillus	-0.058	-0.405	-0.055	-0.007
g_Eggerthella	-0.047	-0.325	-0.019	-0.003
g_Erysipelotrichaceae_incertae_sedis	-0.023	-0.172	-0.017	-0.002
g_Fusobacterium	-0.001	0.032	0.005	0.000
g_Intestinibacter	-0.012	-0.100	-0.004	-0.001
g_Lachnospira	0.023	0.160	0.044	0.006
g_Lachnospiracea_incertae_sedis	-0.028	-0.168	-0.036	-0.005
g_Megasphaera	0.001	0.006	-0.018	-0.003
g_Terrisporobacter	-0.008	-0.016	-0.054	-0.008
g_Veillonella	0.007	-0.011	-0.010	-0.003
g_Weissella	0.025	0.258	-0.029	-0.001

LS1–4, lumbar spines 1–4; BMD, bone mineral density; FN, femoral neck; p, phylum; g, genus.

The ridge regression model was adjusted for age, body mass index, sex, smoking, alcohol drinking, coffee drinking and dietary habits; and statistical significance was indicated by ^*^0.01 ≤ P value < 0.05, and ^**^P value < 0.01.

### Functional Pathway Predictions for the Identified Osteoporosis-Related Gut Microbiota

**Figure 5:**
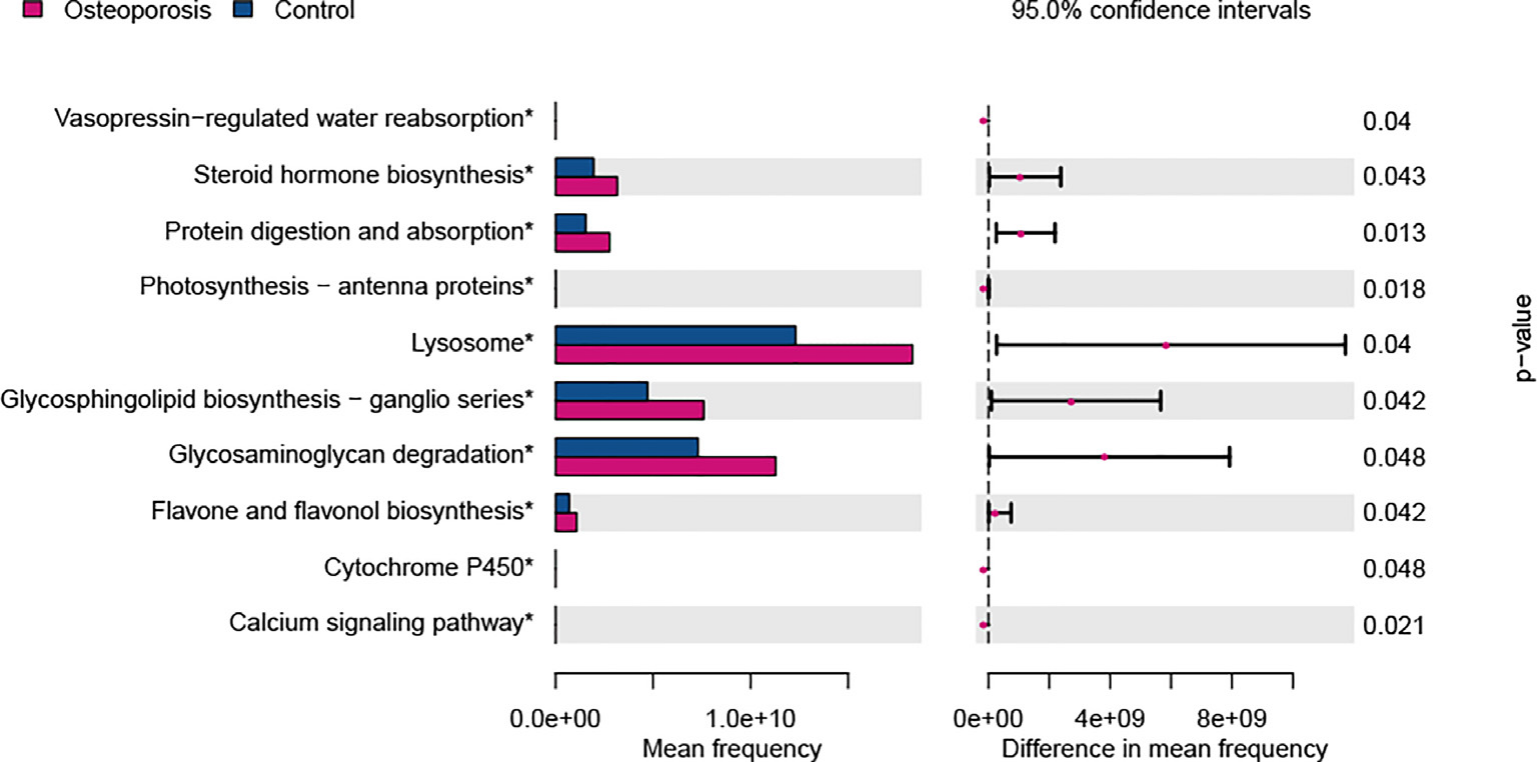
Predicted functional differences between osteoporosis and control groups. A total of 10 metabolic pathways varied between the two groups. Tests were conducted at Kyoto Encyclopedia of Genes and Genomes (KEGG) hierarchical level 3. Difference in mean frequency = mean abundance in osteoporosis group minus mean abundance in control group.

The KEGG functional pathways were predicted with PICRUSt to elucidate the potential roles of gut microbiota identified in this study. As shown in **Figure 5**, 10 KEGG pathways were predicted to show differences between osteoporosis and control groups. Specifically, pathways relevant to steroid hormone biosynthesis, protein digestion and absorption, lysosome, glycosphingolipid biosynthesis, glycosaminoglycan degradation, and flavone and flavonol biosynthesis were functionally enhanced in patients with osteoporosis in comparison with the controls (*P* value < 0.05).

## Discussion

In this study, the 16S rRNA gene sequencing technique was used to quantify gut microbiota compositions from the absolute and relative views. Representative indices for microbial abundances were analyzed to investigate associations of microbial compositions and osteoporosis risk among the Han Chinese elderly. To the best of our knowledge, this is the first osteoporosis-related gut microbiota association study that considers absolute quantifications. Several phylum- and genus-level taxonomic differences were discovered between osteoporosis patients and the controls. With potential confounders controlled, the Bacteroidetes phylum and the *Bacteroides*, *Lactobacillus*, *Eisenbergiella*, and *Eggerthella* genera are associated with risk of osteoporosis. In addition, the *Bacteroides* genus is associated with BMD at the lumbar spine, and the *Anaerovorax* genus is associated with BMD at the femoral neck in adjustment of multiple covariates. Moreover, 10 pathways relevant to steroid hormone biosynthesis, protein digestion and absorption, lysosome, glycosphingolipid biosynthesis, glycosaminoglycan degradation, and flavone and flavonol biosynthesis were predicted on the basis of the KEGG to be functionally enhanced in patients with osteoporosis compared with the controls.

The relative abundance of the *Parabacteroides*, *Flavonifractor*, and *Raoultella* genera had between-group difference, whereas the absolute abundance did not. The enrichment of taxa in relative abundance does not necessarily indicate the alternations in absolute abundance (Props et al., 2017). Different microbial loads in samples may contribute to the discordant enrichment of taxa in relative and absolute abundances. Vandeputte et al. utilized relative and absolute microbiome profiles to assess Crohn’s disease-related microbiome signals, concluding that the microbial load is a key driver of the observed disease-related microbiota alterations (Vandeputte et al., 2017). Several studies focused on the association between gut microbiota composition, expressed in proportional abundances of taxa, and bone health and showed inconsistent results (Wang et al., 2017; Das et al., 2019; Li et al., 2019). Consistent with our findings, one cohort study including 181 participants revealed that the relative abundance of the *Eggerthella* genus was increased in osteoporosis cases (Das et al., 2019). By contrast, Wang et al. reported a reduction in Bacteroidetes phylum in osteoporosis cases (Wang et al., 2017). Caution must be taken in interpreting these results, which were solely observed from relative abundance comparisons, because relative abundance alternations cannot always reflect precise absolute abundance changes (Smets et al., 2016).

The patients with osteoporosis were found to have increased absolute abundances of the Bacteroidetes phylum and *Bacteroides* genus; moreover, *Bacteroides* showed a negative correlation with the BMD and T-score at the lumbar spine. The Bacteroidetes phylum consists of various gram-negative bacteria in the gastrointestinal tract, including *Bacteroides* genus (Eckburg et al., 2005). Lipopolysaccharide (LPS), a component of the gram-negative bacterial outer membrane, can stimulate the production of pro-inflammatory cytokines, resulting in systemic inflammations (Maldonado et al., 2016; Shen et al., 2018). In vivo and *in vitro* studies have suggested that the LPS-induced pro-inflammatory cytokines are involved in the processes of osteoclast formation and bone destruction (Abu-Amer et al., 1997; Zou and Bar-Shavit, 2002; Mörmann et al., 2008). Several observational studies have also reported the inflammation mechanism underlying osteoporosis and/or osteopenia pathogenesis (Scheidt-Nave et al., 2001; Sponholtz et al., 2014). Increasing gram-negative bacteria, such as *Bacteroides*, may trigger a cascade of inflammatory responses that contribute to the initiation of bone loss, ultimately disrupting bone health.

Three genera (*Anaerovorax*, *Lactobacillus*, and *Eisenbergiella*) belonging to the Firmicutes phylum were found to be associated with BMD and osteoporosis risk in this study. The *Anaerovorax* positively correlated with the BMD at the femoral neck. However, high amounts of *Lactobacillus* and *Eisenbergiella* were observed in the osteoporosis group. *Lactobacillus* is commonly used as a probiotic; increased abundance of some *Lactobacillus* species, such as *L.reuteri*, have been reported to prevent bone loss (Nilsson et al., 2018). Our finding of the increased absolute abundance of *Lactobacillus* in patients with osteoporosis suggested that the effect of Lactobacillus on bone metabolism may be species and strain specific. A previous study focusing on weight gain and *Lactobacillus* also indicated that the effect on metabolism varied among different species (Million et al., 2012). A vaginal microbial community research indicated *L.iners* contains features of probiotic *Lactobacillus* as well as of vaginal pathogens (Petrova et al., 2017). Further studies are needed to clarify the role of *Lactobacillus* species and strain on bone health. To the best of our knowledge, no previous studies have reported the association between *Anaerovorax*, and *Eisenbergiella* with bone health whether in humans or animal models. Therefore, further studies are warranted to elucidate the roles of these bacteria in the development of osteoporosis.

In addition, compared with the controls, the patients with osteoporosis exhibited increased abundance of *Eggerthella* genus, which is consistent with the findings of a previous study (Das et al., 2019). Many studies have reported the contributable effect of *Eggerthella* on inflammatory diseases, including rheumatoid arthritis, ankylosing spondylitis and systemic lupus erythematosus (Scher et al., 2013; Chen et al., 2016; He et al., 2016). Specifically, *Eggerthella* was also found to enrich in the vitamin D receptor (VDR) knockout (Vdr^-/-^) mice compared with wide-type mice (Jin et al., 2015). VDR has been previously shown to increase the formation and decrease the resorption of bone, and VDR-mediated activity in osteoblasts and osteocytes can prevent bone loss caused by vitamin D deficiency (Gardiner et al., 2000; Lam et al., 2014). In an epidemiology study, *VDR* gene polymorphisms were found to be significantly associated with the decrease in BMD and increase in osteoporosis risk (He et al., 2015; Kow et al., 2019). The elevated abundance of *Eggerthella* was probably relevant to the inefficient function of vitamin D receptors in the osteoporosis cases.

Functional predictions for gut microbiota revealed that several KEGG pathways may contribute to osteoporosis pathogenesis. We observed that glycosaminoglycan (GAGs) degradation increased in the osteoporosis group. As important extracellular matrix components in bone, GAGs play important roles in regulating biological processes in bone (Salbach et al., 2012). A pilot study in rats presented a positive effect of GAGs on bone formation in a critical bone size defect. In vitro studies demonstrated that GAGs may contribute to bone homeostasis by direct interactions with bone-regulating proteins and cytokines, such as RANKL, OPG, and cathepsin K (Li et al., 2002; Théoleyre et al., 2006). The roles of steroid hormones (estrogen, corticosteroids, androgen, and progesterone) in bone cell development and in the maintenance of normal bone architecture have been well established (Bland, 2000). A recent study revealed that sex steroid deficiency-related bone loss is microbiota dependent (Li et al., 2016). Moreover, great intakes of flavonols and flavones were associated with increased bone density in humans (Zhang et al., 2014). Mechanistic studies indicated that flavonoid may regulate bone metabolism though the inhibition of RANKL-induced osteoclast differentiation (Lee et al., 2009).

This study is the first to investigate the composition alternations of osteoporosis-related gut microbiota considering the absolute and relative abundance of microbiomes. In the relative abundance comparisons, data obtained from our previous work applying a same laboratory sequencing platform were combined to increase the statistical power. Spiked exogenous sequences of known concentrations were applied to quantify the absolute abundance of gut microbiota in the present study. Another group of methods, such as flow cytometry, total DNA, quantitative PCR, or digital PCR, can also be used for microbial taxa quantification (Kleyer et al., 2017; Vandeputte et al., 2017; Contijoch et al., 2019; Barlow et al., 2020). The relative abundances of microbial taxa were transformed to absolute data by measuring the total concentration of cells, DNA, or amplicons for flow cytometry, total DNA, and quantitative PCR, respectively. The digital PCR quantitative microbial analysis is appropriate for biogeographically diverse sample types and enables the mapping of the microbial biogeography of the gastrointestinal tract.

## Study Limitations

Several limitations should be acknowledged. First, the present study is of a case control design, which has a limitation in causality inference from altered microbiota composition to osteoporosis prevalence risk. Second, a limitation in our method is that 16S rRNA gene sequencing does not have sufficient resolution to identify the microbiota at the species or strain level, and thus can lead to the omission of some microbial taxa (Ravi et al., 2018). Third, dietary factors are crucial in driving microbial community structure, as well as lifestyle (e.g., physical exercise and stress) and genetic factors (Conlon and Bird, 2014; Wang et al., 2016). Although a few covariates were adjusted, residual confounding by these unmeasured factors may influence the results to some extent. Fourth, ethnicity and residence location of subjects were also associated with variations of microbial abundance (Gupta et al., 2017; He et al., 2018). In this study, the participants were all Han Chinese from the same region, possibly affecting the generalizability of our findings to other ethnic and regional populations. Therefore, future studies in this filed should consider the dietary, lifestyle, and genetic factors and recruit participants from various regions.

## Conclusion

Through absolute quantification 16S rRNA gene sequencing, this study suggests that osteoporosis and bone density at the lumbar spine and femoral neck of Han Chinese elderly are potentially associated with the altered composition of gut microbiota at the phylum and genus levels. In particular, our findings indicate a link between Bacteroidetes-dominated microbiome and osteoporosis risk. The findings of this study may provide new clues to understand the microbiota-related mechanism in osteoporosis pathogenesis and provide potential biomarkers and therapeutic targets for disease prevention and treatment in the future.

## Data Availability Statement

The data presented in the study are deposited in the National Center for Biotechnology Information (NCBI) Bioproject database with accession number PRJNA724901, https://www.ncbi.nlm.nih.gov/sra/PRJNA724901.

## Ethics Statement

The studies involving human participants were reviewed and approved by Institutional Review Board of Tongji Medical College, Huazhong University of Science and Technology. The patients/participants provided their written informed consent to participate in this study.

## Author Contributions

QW and QH designed and managed the research. MW, QW, and CL contributed to the statistical analysis and manuscript writing. CL, MW, YD, HZ, YC, and YZ contributed to the data collection. All authors contributed to the article and approved the submitted version.

## Funding

The work was funded by the National Natural Science Foundation of China [Grant no. 81573235], the Natural Science Foundation of Hubei Province [Grant nos. 2019CFB647 and 2019CFB709], and the Fundamental Research Funds for the Central Universities [Grant no. 2019kfyXKJC003].

## Conflict of Interest

The authors declare that the research was conducted in the absence of any commercial or financial relationships that could be construed as a potential conflict of interest.
