# Supplementary material for: High-Throughput Absolute Quantification Sequencing Revealed Osteoporosis-Related Gut Microbiota Alterations in Han Chinese Elderly
Source: Front Cell Infect Microbiol. 2021 Apr 30;11:630372. doi: 10.3389/fcimb.2021.630372 (PMC8120270; doi:10.3389/fcimb.2021.630372)
Supplement: Supplementary file 1 [file DataSheet_1.pdf]

**High-throughput absolute quantification sequencing revealed osteoporosis-related gut microbiota alterations in Han Chinese elderly**

Muhong Wei[1], Can Li[1], Yu Dai[3], Haolong Zhou[1], Yuan Cui[1], Yun Zeng[4], Qin Huang[2,*], Qi Wang[1,*]

1. MOE Key Lab of Environment and Health, Department of Epidemiology and Biostatistics, School of Public Health, Tongji Medical College, Huazhong University of Science and Technology, Wuhan 430030, China

2. Department of Rehabilitation Medicine, Union Hospital, Tongji Medical College, Huazhong University of Science and Technology, Wuhan 430030, China

3. Department of Nuclear Medicine, Union Hospital, Tongji Medical College, Huazhong University of Science and Technology, Wuhan 430030, China

4. Wuhan NO.1 Hospital, Wuhan 430030, China

**Methods**

**Absolute abundance calculation**

Taken the data of one participant (D0004) for example to introduce the calculation of the absolute abundance based on the spike-in sequences. Two-step work is performed for the calculation.

1. Standard curve creation

The standard curve was drew based on the numbers of read (17964, 18333, 2267, 3035, 218, 162, 31, 40, 22) and of corresponding copy ($1.82 \times 10^6$, $1.82 \times 10^6$, $1.82 \times 10^5$, $1.82 \times 10^5$, $1.82 \times 10^4$, $1.82 \times 10^4$, $1.82 \times 10^3$, $1.82 \times 10^3$, $1.82 \times 10^3$) of the nine spike-in sequences. The standard curve and corresponding linear equation ($y = 0.9437x - 1.6303$; y equals to log10 (OTU reads), and x equals to log10 (copies)) were presented in Supplementary Fig. 1A.

2. Number of OTU copy estimate for the sample

The corresponding numbers of copy of the top 10 OTUs (OTU109, OTU11, OTU102, OTU308, OTU118, OTU106, OTU126, OTU158, OTU74, and OTU135) in the D0004 sample were estimated referring to the standard curve obtained in the first step. The numbers of read of the top 10 OTUs were 26193, 20626, 20514, 9261, 7579, 5859, 5670, 4642, 4169, and 3704, respectively. And referring to the linear equation for the standard curve (Supplementary Fig. 1A), corresponding numbers of OTU copy can be estimated from the reads of OTUs, equaling to 2566437, 1992368, 1980906, 852836, 689647, 525012, 507083, 410221, 366067, 322950 copies, respectively (Supplementary Fig. 1B).

**Supplementary Table 1.** Characteristics of included participants in this study

| | Absolute profiling | | | Relative profiling | | |
|---|---|---|---|---|---|---|
| | Osteoporosis (n = 44) | Control (n = 64) | *P* value | Osteoporosis (n = 74) | Control (n = 101) | *P* valve |
| Age (years, mean ± SD) # | 69.73 ± 5.47 | 67.84 ± 4.83 | 0.061 | 68.59 ± 5.36 | 67.06 ± 5.00 | 0.054 |
| Female, n (%)& | 35 (79.5) | 32(50.0) | 0.002 | 57(77.0) | 48(47.5) | <0.001 |
| BMI (kg/$m^2$) # | 22.60 ± 2.79 | 24.43 ± 3.25 | 0.003 | 22.81 ± 2.97 | 24.53 ± 3.15 | <0.001 |
| Smoking, n (%)& | 5 (11.4) | 9 (14.1) | 0.682 | 7 (9.5) | 14 (13.9) | 0.376 |
| Alcohol drinking, n (%)& | 6 (13.6) | 12 (18.8) | 0.484 | 7 (9.5) | 16 (15.8) | 0.217 |
| Coffee drinking, n (%)& | 7 (16.3) | 6 (9.4) | 0.284 | 10 (13.7) | 10 (9.9) | 0.438 |
| Dietary habits, n (%)& | | | 0.161 | | | 0.161 |
| Omnivores | 34 (77.3) | 56 (87.5) | | 34 (45.9) | 56 (55.4) | |
| Vegetarians | 10 (22.7) | 8 (12.5) | | 10 (13.5) | 8 (7.9) | |
| Prevalent fracture, n (%)& | 24 (54.5) | 14 (22.6) | 0.001 | 36 (48.6) | 21 (21.2) | <0.001 |
| LS1–4 BMD (g/$cm^2$, mean ± SD)# | 0.87 ± 0.13 | 1.09 ± 0.14 | <0.001 | 0.87 ± 0.12 | 1.11 ± 0.16 | <0.001 |
| LS1–4 Z-score (mean ± SD)# | -0.75 ± 1.09 | 0.56 ± 1.06 | <0.001 | -0.84 ± 1.04 | 0.65 ± 1.19 | <0.001 |
| LS1–4 T-score (mean ± SD)# | -2.64 ± 1.00 | -0.77 ± 1.11 | <0.001 | -2.65 ± 0.97 | -0.60 ± 1.25 | <0.001 |
| FN BMD (g/$cm^2$, mean ± SD)# | 0.67 ± 0.10 | 0.84 ± 0.11 | <0.001 | 0.69 ± 0.10 | 0.86 ± 0.11 | <0.001 |
| FN Z-score (mean ± SD)# | -0.59 ± 1.84 | 0.16 ± 0.77 | <0.001 | -0.58 ± 1.48 | 0.26 ± 0.82 | <0.001 |
| FN T-score (mean ± SD)# | -2.66 ± 0.81 | -1.35 ± 0.78 | <0.001 | -2.39 ± 1.10 | -1.14 ± 0.92 | <0.001 |

BMI, body mass index; LS1–4, lumbar spines 1–4; BMD, bone mineral density; FN, femoral neck; SD, standard deviation.

&Chi-square test was used for statistical analysis.

#Two independent-sample *t* test was used for statistical analysis.

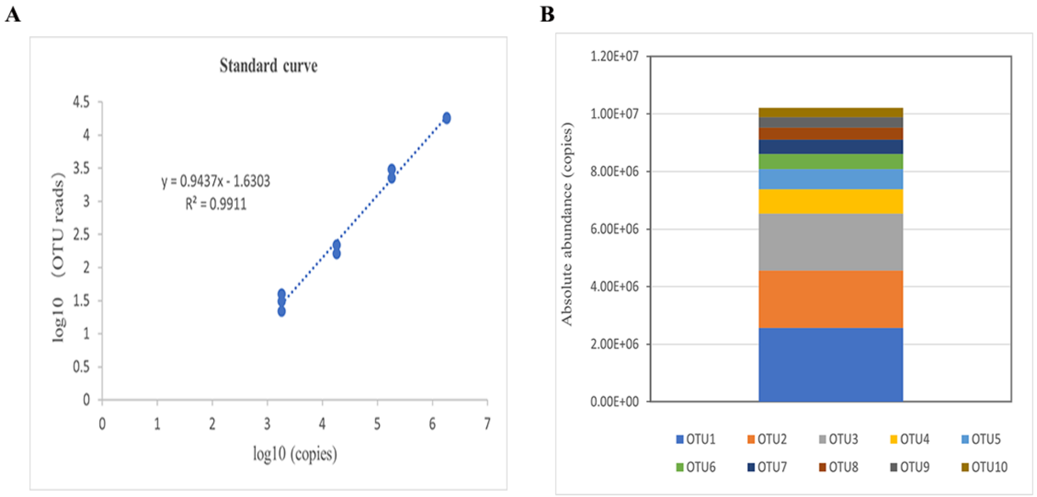


**Supplementary Figure 1.** Example for absolute abundance calculation based on the spike-in sequences.
